# Comparative Quantification of Fungiform Papillae Density and Taste Perception in Anemic and Healthy Controls: A Case-Control Study

**DOI:** 10.7759/cureus.67082

**Published:** 2024-08-17

**Authors:** Krishna Verma, Ramya Ramadoss, Sandhya Sundar, Suganya Panneer Selvam, Hemashree K

**Affiliations:** 1 Oral Biology, Saveetha Institute of Medical and Technical Sciences, Chennai, IND; 2 Oral Pathology and Oral Biology, Saveetha Institute of Medical and Technical Sciences, Chennai, IND; 3 Oral Pathology, Saveetha Institute of Medical and Technical Sciences, Chennai, IND; 4 Oral and Maxillofacial Pathology, Saveetha Institute of Medical and Technical Sciences, Chennai, IND

**Keywords:** taste strips, nutrition, fungiform papillae, taste perception, anemia

## Abstract

Background

Taste perception is crucial for dietary choices, nutrition, and overall health. The human tongue, particularly the fungiform papillae, plays a significant role in taste sensation, especially for sweet and umami flavors. Anemia, a common condition characterized by low hemoglobin levels, can affect sensory perception, including taste. Recent research has begun to explore the relationship between fungiform papillae density and taste perception in individuals with anemia.

Objective

This study aimed to investigate the comparative quantification of fungiform papillae density and its correlation with taste perception in anemic and healthy individuals, with the goal of understanding the underlying mechanisms linking anemia to taste disturbances.

Methods

A total of 100 adults (50 anemic and 50 healthy controls) aged 18-65 participated in the study. Demographic data, dietary habits, and medical history were collected. Taste perception was assessed using a taste strip test and psychophysical scaling methods, including magnitude estimation and the method of constant stimuli. Fungiform papillae density was quantified using high-resolution images of the tongue. Statistical analyses, including t-tests and correlation analyses, were conducted to compare the groups and explore relationships between hemoglobin levels, papillae density, and taste perception.

Results

The study found that anemic individuals had a slightly lower mean fungiform papillae density (49 papillae per square centimeter) compared to healthy controls (57 papillae per square centimeter). In the taste strip test, anemic participants showed reduced accuracy in taste perception, particularly for bitter flavors. However, psychophysical scaling results, as measured by magnitude estimation, revealed no statistically significant difference in subjective taste intensity between the anemic and healthy control groups (p=0.8666).

Conclusion

The study suggests that while anemic individuals may exhibit reduced fungiform papillae density and altered taste perception, particularly for bitter flavors, the overall subjective perception of taste intensity does not significantly differ from that of healthy controls. These findings contribute to the understanding of taste disturbances in anemia and may inform future diagnostic and therapeutic approaches for managing taste disorders in affected individuals

## Introduction

Taste perception is integral to the human sensory experience and is intimately tied to dietary preferences, nutritional intake, and overall health [1]. Within the oral cavity, the human tongue is the primary organ for detecting taste sensations, housing specialized taste buds within its intricate papillary structures [1]. Among these, the fungiform papillae stand out for their mushroom-like morphology and role in hosting taste buds sensitive to sweet and umami flavors [2].

Anemia, a prevalent hematological disorder characterized by low levels of red blood cells or hemoglobin, affects systemic physiology and sensory perception [3,4]. Taste disturbances, such as dysgeusia and hypogeusia, have been reported in individuals with anemia, posing challenges to dietary habits and nutritional status [5]. Recent studies have begun to explore the potential connection between fungiform papillae density and taste perception, particularly in the context of anemia [2,5]. Investigating this relationship offers valuable insights into the complex interplay between oral morphology and sensory function, with implications for understanding taste disorders in individuals with anemia.

The process of taste perception extends beyond surface sensations, involving intricate molecular pathways and sensory integration mechanisms [6]. Taste receptors within taste buds detect specific molecules and transmit signals to the brain, contributing to the overall flavor experience [7]. However, the molecular mechanisms governing the maintenance and regeneration of papillae and taste buds remain areas of ongoing research [5,6].

Despite the growing recognition of taste disturbances in anemic individuals, the precise relationship between fungiform papillae density and altered taste perception remains underexplored. While some studies have hinted at a possible connection between oral morphology and sensory function, the specific role of papillary density in mediating taste changes in anemia has not been thoroughly investigated [5,6]. Furthermore, the molecular mechanisms that could explain the maintenance, regeneration, and functional changes of taste buds in the context of anemia are still not well understood. This research gap highlights the need for a detailed comparative analysis to clarify the association between fungiform papillae density, taste perception, and anemia, which could have significant implications for improving diagnostic and therapeutic strategies for taste disorders in affected individuals.

This study investigates the comparative quantification of fungiform papillae density and its correlation with taste perception in both anemic and healthy individuals. By examining potential differences in papillary morphology and taste sensitivity between these groups, we seek to elucidate the underlying mechanisms linking anemia with taste disturbances. Also, this study endeavors to unravel the complex relationship between fungiform papillae density, taste perception, and anemia. Through a comprehensive comparative analysis, we aim to shed light on the mechanisms underlying taste disturbances in individuals with anemia, paving the way for targeted diagnostic and therapeutic interventions.

## Materials and methods

A total of 100 individuals participated in this study, comprising 50 patients diagnosed with anemia and 50 healthy controls. The sample size was determined using G*Power analysis to ensure adequate statistical power. Anemic participants were selected based on clinical parameters, specifically hemoglobin levels below the normal range for their age and sex. Healthy controls were recruited from individuals with normal hemoglobin levels and no known hematological disorders. Inclusion criteria for both groups required participants to be adults aged 18-65, with no chronic diseases or conditions affecting taste perception, such as smoking, oral infections, or medications known to alter taste. Exclusion criteria included pregnancy, lactation, recent upper respiratory infections, and known allergies to the taste solutions used in the study. The study adhered to ethical guidelines and received approval from the Institutional Review Board of Saveetha Dental College (approval number: SRB/SDC/OBIO-2214/24/120). Informed consent was obtained from all participants, and confidentiality measures were strictly followed throughout the study.

Demographic details

The study included 100 participants, divided equally into two groups: those diagnosed with anemia and healthy controls. Demographic characteristics such as age, sex, ethnicity, dietary habits, and medical history were recorded for each participant. Both groups had a similar age range, with a balanced representation of males and females. The majority of participants in both groups were of Asian ethnicity, with a small number from other ethnic backgrounds. Dietary habits varied slightly, with a minority in each group identifying as vegetarians, while the majority were non-vegetarians. Most participants had no significant medical history, with a few reporting minor conditions.

Taste strip test

Taste strips representing sweet, sour, and bitter tastes were prepared in-house by saturating paper strips with corresponding solutions and uniformly baking them in an oven to ensure consistent taste delivery [8]. Participants were provided with annotated images of the tongue delineating distinct regions to help them identify the specific area where taste sensations were most pronounced during the taste perception task. Taste strips were presented in a randomized sequence, with participants rinsing their mouths thoroughly between each presentation to minimize carryover effects.

Quantification of the fungiform papillae

High-resolution images of the tongues of both anemic and healthy control participants were captured to facilitate the physical counting of the fungiform papillae. A standardized imaging protocol ensured accurate and reliable measurements. The manual counting protocol involved capturing images using a digital camera and dividing the tongue into specific regions, including the anterior, middle, posterior, and sides. Trained observers meticulously counted the fungiform papillae in each area, avoiding double-counting or missing papillae. Data on papillary counts were recorded systematically, and quality control measures, including independent counting by a second observer, were implemented to assess inter-observer reliability. Statistical analysis, including t-tests or non-parametric equivalents, was performed to compare papillary density between the two groups.

Psychophysical scaling

Psychophysical scaling techniques were utilized to precisely assess taste perception in the study. The magnitude estimation method involved presenting participants with reference taste stimuli of known intensity and asking them to assign numerical values to other taste stimuli based on their perceived intensity. For instance, a sweet reference solution with an intensity rating of 100 units was used, and participants rated additional stimuli such as bitter or sour relative to this reference. A bitter stimulus perceived as twice as intense as the reference might be rated as 200 units, while a less intense sour stimulus could be rated as 50 units. The method of constant stimuli was also employed, where participants were presented with a range of taste stimuli varying in intensity, such as sweet solutions from 0.1% to 1.0% concentrations. Participants rated these stimuli on a scale from 0 (no taste) to 100 (maximum intensity), with a 0.1% solution possibly rated as 20 units and a 1.0% solution as 80 units. These methods provided quantitative data on subjective taste perceptions, complementing traditional statistical analyses and offering a detailed understanding of taste sensitivity in both anemic and healthy control groups.

Statistical analysis

Descriptive statistics were used to summarize demographic data and hemoglobin concentrations. Inferential statistics using t-tests were employed to assess differences in fungiform papillae density and taste perception between anemic and healthy control groups. Correlation analyses were conducted to explore the relationship between hemoglobin levels, fungiform papillae density, and taste perception scores. Significance levels were set at p<0.05. 

## Results

The study included a total of 100 participants, divided into two groups: 50 individuals diagnosed with anemia and 50 healthy controls. Demographic characteristics such as age, sex, ethnicity, dietary habits, and medical history were systematically recorded for each participant. The mean age of the anemic group was 43.1 years (SD±7.2), compared to 40.5 years (SD±6.1) in the healthy control group, with both groups ranging in age from 18 to 65 years. The sex distribution in the anemic group consisted of 23 males (46%) and 27 females (54%), while the healthy control group included 29 males (58%) and 21 females (42%). The majority of participants across both groups were of Asian ethnicity (92%), with a small proportion (8%) representing other ethnicities. Dietary habits were noted, with 10 participants (20%) in the anemic group and eight participants (16%) in the healthy control group identifying as vegetarians, while the remaining participants were non-vegetarians (80% in the anemic group and 84% in the healthy control group). Medical history data revealed that 35 participants (70%) in the anemic group and 37 participants (74%) in the healthy control group had no significant medical history, with the remaining participants having a history of minor conditions.

**Figure 1:**
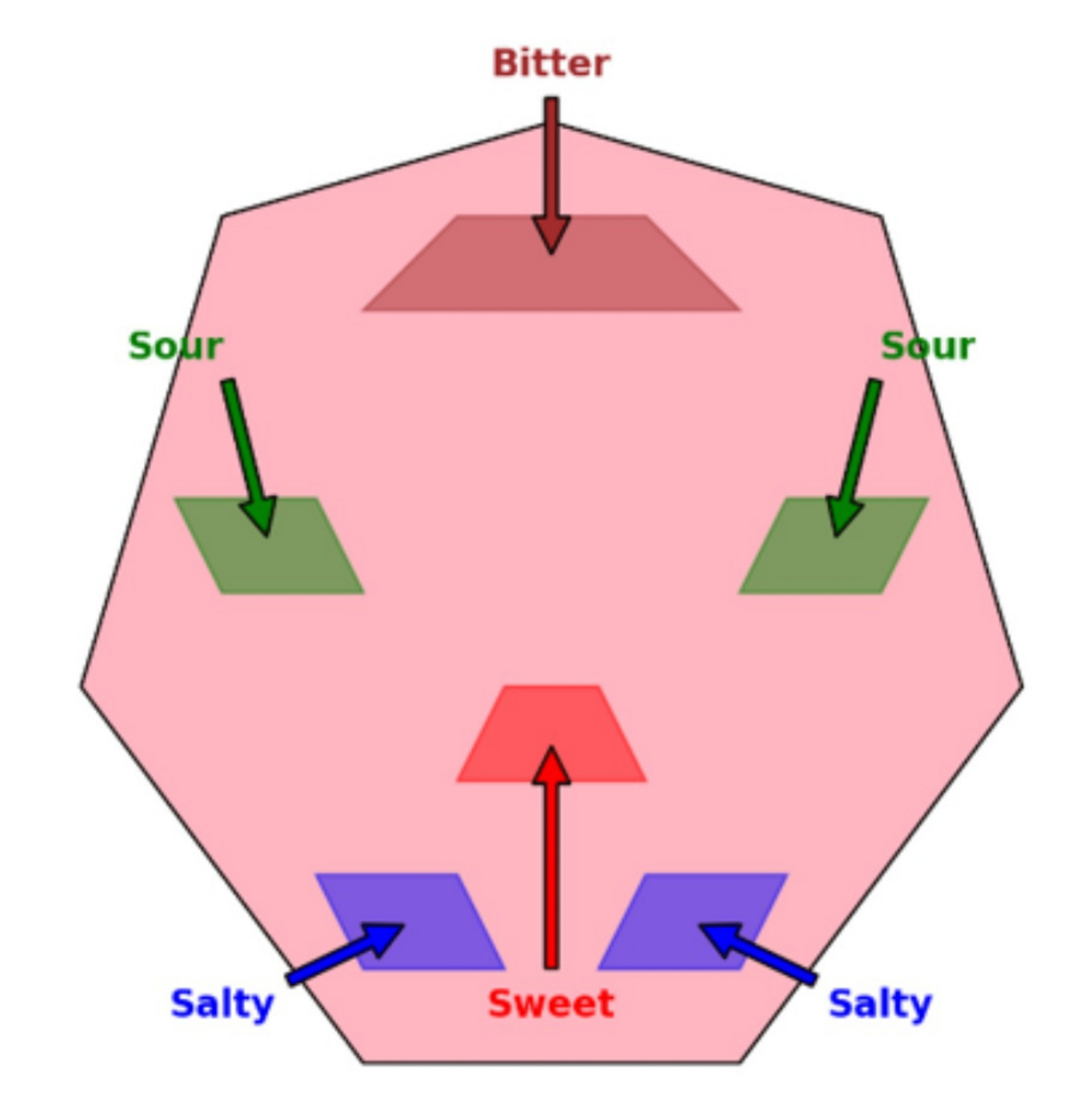
Taste mapping of the tongue This image has been drawn by the author

In our study, participants were administered taste strips and instructed to identify the region on a sheet of paper that exhibited the most pronounced taste sensation (Figure 1). The findings revealed that individuals suffering from anemia displayed reduced accuracy in perceiving the taste of the taste strips in comparison to those who were not afflicted with the condition (Table 1). The anemic participants experienced a generalized taste sensation primarily localized in the central part of their tongue. At the same time, they also struggled to perceive the bitter taste, which was readily discernible to the standard control group.

**Table 1:** Description regarding the demographic details and hemoglobin concentration of the study groups A p-value less than 0.05 is considered significant

Variable	Anemic group (n=50)	Healthy control (n=50)	Total (n=100)
Age (years)			
Mean±SD	43.1±7.2	40.5±6.1	41.8±6.8
Range	18-65	18-65	18-65
Sex			
Male (n, %)	23 (46%)	29 (58%)	52 (52%)
Female (n, %)	27 (54%)	21 (42%)	48 (48%)
Ethnicity			
Asian (n, %)	46 (92%)	46 (92%)	92 (92%)
Other (n, %)	4 (8%)	4 (8%)	8 (8%)
Dietary habits			
Vegetarian (n, %)	10 (20%)	8 (16%)	18 (18%)
Non-vegetarian (n, %)	40 (80%)	42 (84%)	82 (82%)
Medical history			
No significant history (n, %)	35 (70%)	37 (74%)	72 (72%)
History of minor conditions (n, %)	15 (30%)	13 (26%)	28 (28%)

Quantification of fungiform papillae density

The analysis comparing the quantification of fungiform papillae density between healthy individuals and those diagnosed with anemia revealed significant differences. Across both groups, the mean fungiform papillae density was slightly lower in individuals diagnosed with anemia compared to healthy controls. Specifically, healthy participants exhibited an average papillary density of 57 papillae per square centimeter, whereas anemic individuals had a slightly reduced mean density of 49 papillae per square centimeter.

Psychophysical scaling result

**Figure 2:**
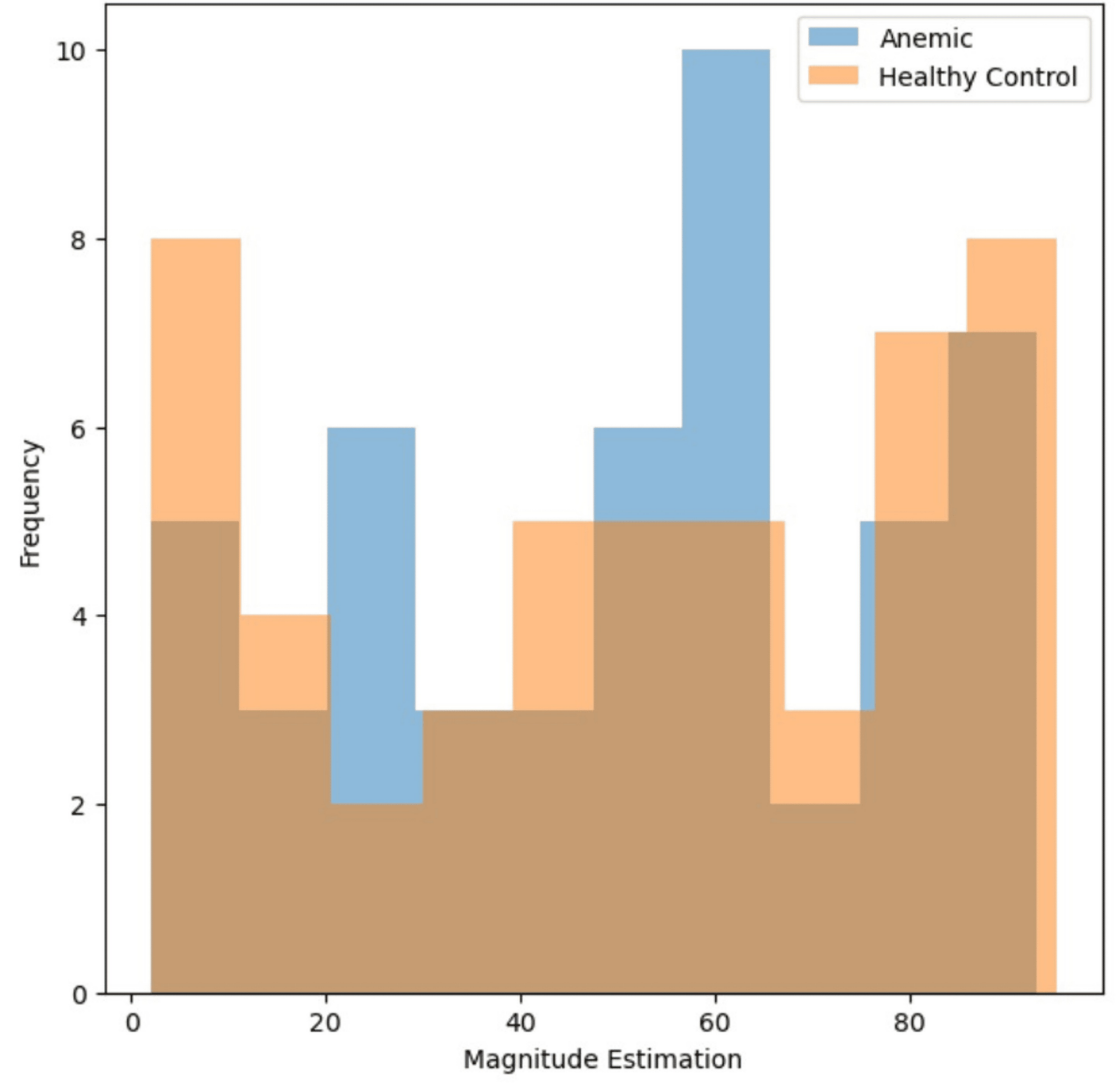
Distribution of magnitude effect

A histogram comparison plot (Figure 2) was generated to visually depict the distribution of "Magnitude Estimation" values for both groups. From the plot, it is evident that the "Anemic" group tends to have lower "Magnitude Estimation" values compared to the "Healthy Control" group.

**Figure 3:**
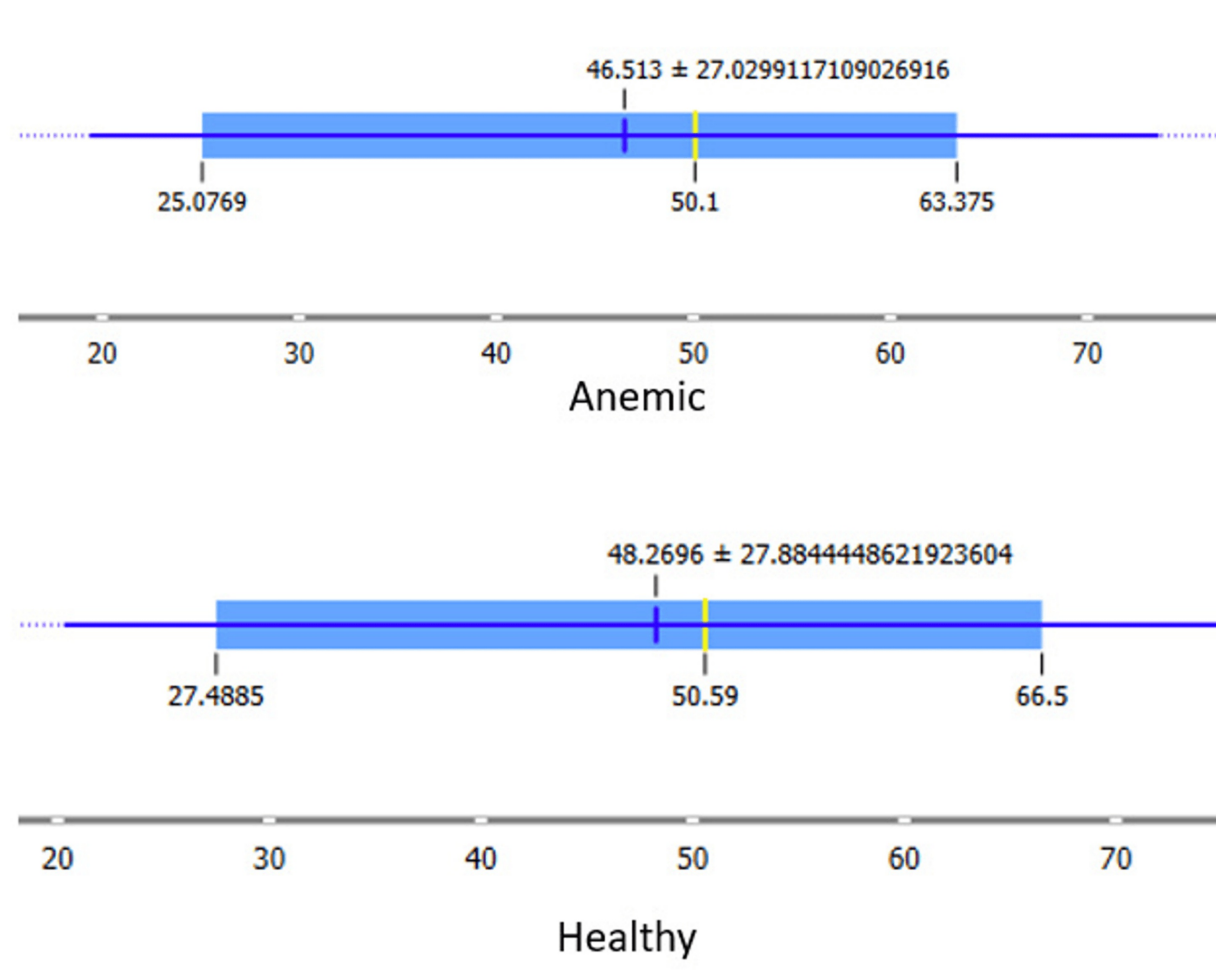
Mean scoring between the normal and anemic groups

A two-sample t-test was conducted to quantitatively assess the difference in "Magnitude Estimation" between the two groups. The calculated t-statistic was found to be -0.17, indicating that the means of the "Anemic" and "Healthy Control" groups are quite similar. Additionally, the p-value associated with the t-test was determined to be 0.8666, significantly higher than the conventional significance level of 0.05. Therefore, based on the statistical analysis, it can be concluded that there is no statistically significant difference in "Magnitude Estimation" between the anemic and healthy control participants in this dataset (Figure 3).

These findings suggest that, despite the observed differences in the histogram plot, the subjective perception of taste intensity, as measured by "Magnitude Estimation," does not significantly differ between individuals with anemia and those without hematological disorders.

## Discussion

Taste mapping in anemic patients involves evaluating taste perception across different regions of the tongue systematically. The aim is to identify potential alterations in taste sensitivity and preferences. Through standardized methods and stimuli representing various taste modalities, such as sweet, sour, salty, and bitter, researchers can create a map showing the distribution and intensity of taste perception. This mapping process allows identifying specific areas on the tongue where taste perception may be heightened or diminished compared to healthy controls. Furthermore, taste mapping studies in anemic patients provide valuable insights into the underlying mechanisms contributing to taste alterations and inform targeted interventions to improve taste perception and dietary intake in affected individuals [2-4].

The pathogenesis of taste perception alterations in anemia involves complex physiological mechanisms. The alterations are primarily driven by iron deficiency, a prevalent cause of anemia. Insufficient iron levels compromise taste bud structure and function, impairing their ability to detect and transmit taste signals [5]. Consequently, it leads to changes in taste perception. Additionally, iron deficiency disrupts neurological pathways involved in taste perception, affecting the synthesis of neurotransmitters crucial for taste signal transmission [6]. Studies indicate that anemic individuals may exhibit reduced sensitivity to sweet tastes and heightened sensitivity to bitter tastes [7].

Secondary factors associated with anemia, such as inflammation and oxidative stress, further exacerbate taste perception disturbances by disrupting taste bud function and neural signaling [8]. These alterations in taste perception significantly impact anemic individuals' food preferences and dietary behaviors, potentially leading to imbalanced nutritional intake. A comprehensive understanding of these intricate mechanisms is paramount for elucidating the pathogenesis of taste perception alterations in anemia and devising targeted interventions to facilitate these changes and enhance nutritional outcomes in affected individuals.

In anemia, changes in the sensitivity and preference for different taste modalities are commonly observed. Anemic individuals may experience variations in the perception of sweet, sour, salty, and bitter tastes, with particular tastes being more affected than others [9]. Anemia-induced alterations often result in reduced perception of sweet tastes, attributed to disruptions in taste bud function and neural signaling pathways caused by underlying nutritional deficiencies, particularly iron deficiency [10]. Iron is essential for the proper functioning of taste receptors and the transmission of taste signals to the brain.

A diminished perception of sweetness is a common observation in anemic individuals [11]. Conversely, heightened sensitivity to bitter tastes is thought to be linked to alterations in taste bud morphology and function resulting from iron deficiency. Moreover, studies suggest that anemia-induced changes in saliva composition and oral microbiota may contribute to the perception of bitter tastes [12,13]. Additionally, alterations in taste perception can influence dietary preferences and food choices, potentially impacting nutritional intake and overall health in anemic individuals.

While alterations in salty and sour tastes are less commonly reported in anemia compared to changes in sweet and bitter tastes, disruptions in taste perception for these modalities may still occur. The exact mechanisms underlying alterations in salty and sour tastes in anemia are not as well understood. Still, they could involve similar pathways affected by nutritional deficiencies and physiological changes associated with the condition [14]. Overall, the alterations in taste perception observed in anemia reflect the complex interplay of various physiological factors, including nutritional deficiencies, changes in taste bud morphology and function, and disruptions in neural signaling pathways. Understanding these mechanisms is essential for developing targeted interventions to address taste disturbances and optimize nutritional status in individuals with anemia.

The role of zinc and other nutrients is critical in maintaining taste bud function, as deficiencies in these nutrients, including zinc, can impair taste perception [15]. Zinc deficiency, often co-occurring with anemia, highlights the need to consider multiple nutritional factors when studying taste alterations in anemia. Psychological and social factors also play a significant role; conditions like anxiety or depression, which can accompany chronic anemia, may influence taste perception and food preferences. Additionally, social factors, such as cultural food practices and socioeconomic status, can further modulate the impact of taste alterations on dietary choices.

Understanding these dynamics has important clinical implications. By mapping taste changes and studying their underlying mechanisms, healthcare professionals can better tailor dietary recommendations and supplementation strategies for anemic patients [16]. This targeted approach can help address specific nutrient deficiencies and manage taste changes, thereby improving patients' adherence to dietary advice and enhancing their overall nutritional status.

The limitation of the study is that taste perception is inherently subjective and can be influenced by various factors, including individual sensitivity, psychological state, and cultural background. This subjectivity complicates the standardization of measurements and consistent interpretation of results across studies. Additionally, many studies on taste perception in anemia, including taste mapping, are cross-sectional, limiting the ability to draw causal inferences. Longitudinal studies are necessary to establish a causal relationship between anemia and taste perception changes over time.

## Conclusions

This study reveals significant differences in fungiform papillae density between anemic and healthy individuals, with notable variations in taste perception, particularly in the accuracy of identifying tastes and reduced sensitivity in anemic patients. Despite a lower fungiform papillae density in anemic individuals, subjective taste intensity, measured through psychophysical scaling, did not show significant differences between the groups. These findings suggest that while anemia affects taste perception and papillary morphology, the subjective experience of taste may be influenced by other factors beyond papillary density. This emphasizes the importance of considering these sensory disturbances when addressing dietary and nutritional challenges in anemic patients. Understanding the underlying mechanisms of these alterations, such as potential changes in taste receptor function or central processing of taste information, can guide the development of targeted interventions. These interventions could include tailored dietary recommendations and taste-enhancing strategies to improve taste perception and overall nutritional status in those affected by anemia. Further research is needed to explore the physiological and biochemical pathways involved in these sensory disturbances and to develop effective approaches for mitigating their impact on patient well-being and quality of life.
